# Atomoxetine and citalopram alter brain network organization in Parkinson’s disease

**DOI:** 10.1093/braincomms/fcz013

**Published:** 2019-09-06

**Authors:** Robin J Borchert, Timothy Rittman, Charlotte L Rae, Luca Passamonti, Simon P Jones, Deniz Vatansever, Patricia Vázquez Rodríguez, Zheng Ye, Cristina Nombela, Laura E Hughes, Trevor W Robbins, James B Rowe

**Affiliations:** 1 Department of Clinical Neurosciences, University of Cambridge, Cambridge, UK; 2 Sackler Centre for Consciousness Science, University of Sussex, Brighton, UK; 3 School of Psychology, University of Sussex, Falmer, UK; 4 Department of Biomedical Sciences, National Research Council, Institute of Bioimaging and Molecular Physiology, Segrate, Italy; 5 Institute of Science and Technology for Brain-inspired Intelligence, Fudan University, Shanghai, PR China; 6 Key Laboratory of Mental Health, Institute of Psychology, Chinese Academy of Sciences, Beijing, China; 7 Department of Biological and Health Psychology, Universidad Autónoma de Madrid, Madrid, Spain; 8 Neurosurgery Department, Hospital Clínico San Carlos, Madrid, Spain; 9 MRC Cognition and Brain Sciences Unit, University of Cambridge, Cambridge, UK; 10 Behavioural and Clinical Neuroscience Institute, University of Cambridge, Cambridge, UK

**Keywords:** Parkinson’s disease, atomoxetine, citalopram, network connectivity, resting-state

## Abstract

Parkinson’s disease has multiple detrimental effects on motor and cognitive systems in the brain. In contrast to motor deficits, cognitive impairments in Parkinson’s disease are usually not ameliorated, and can even be worsened, by dopaminergic treatments. Recent evidence has shown potential benefits from restoring other neurotransmitter deficits, including noradrenergic and serotonergic transmission. Here, we study global and regional brain network organization using task-free imaging (also known as resting-state), which minimizes performance confounds and the bias towards predetermined networks. Thirty-three patients with idiopathic Parkinson’s disease were studied three times in a double-blinded, placebo-controlled counter-balanced crossover design, following placebo, 40 mg oral atomoxetine (selective noradrenaline reuptake inhibitor) or 30 mg oral citalopram (selective serotonin reuptake inhibitor). Neuropsychological assessments were performed outside the scanner. Seventy-six controls were scanned without medication to provide normative data for comparison to the patient cohort. Graph theoretical analysis of task-free brain connectivity, with a random 500-node parcellation, was used to measure the effect of disease in placebo-treated state (versus unmedicated controls) and pharmacological intervention (drug versus placebo). Relative to controls, patients on placebo had executive impairments (reduced fluency and inhibitory control), which was reflected in dysfunctional network dynamics in terms of reduced clustering coefficient, hub degree and hub centrality. In patients, atomoxetine improved fluency in proportion to plasma concentration (*P* = 0.006, *r*^2^ = 0.24), and improved response inhibition in proportion to increased hub Eigen centrality (*P* = 0.044, *r*^2^ = 0.14). Citalopram did not improve fluency or inhibitory control, but its influence on network integration and efficiency depended on disease severity: clustering (*P* = 0.01, *r*^2^ = 0.22), modularity (*P* = 0.043, *r*^2^ = 0.14) and path length (*P* = 0.006, *r*^2^ = 0.25) increased in patients with milder forms of Parkinson’s disease, but decreased in patients with more advanced disease (Unified Parkinson’s Disease Rating Scale motor subscale part III > 30). This study supports the use of task-free imaging of brain networks in translational pharmacology of neurodegenerative disorders. We propose that hub connectivity contributes to cognitive performance in Parkinson’s disease, and that noradrenergic treatment strategies can partially restore the neural systems supporting executive function.

## Teaser

Targeting the deficits in serotonergic and noradrenergic transmission can restore cognitive function in Parkinson’s disease. Borchert *et al.* use resting-state imaging to show that changes in hub connectivity on atomoxetine correlate with improved response inhibition while the potential therapeutic effect of citalopram depends on severity of disease.

## Graphical Abstract

**Graphical Abstract:**
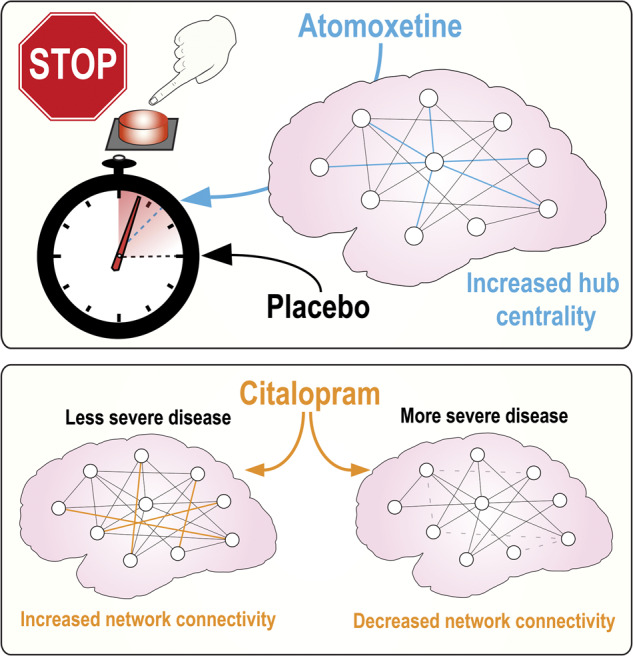

## Introduction

Parkinson’s disease is characterized by its movement disorder but can also cause mild-to-severe cognitive deficits which often involve impaired executive control. Dopaminergic therapies have limited efficacy for the treatment of cognitive changes in Parkinson’s disease and may even worsen impulsivity (Cools *et al.*, 2003; Voon *et al.*, 2006; Weintraub *et al.*, 2006). To restore cognitive function, an alternative approach is to target the deficits in noradrenergic transmission (Williams-Gray *et al.*, 2007; Robbins and Arnsten, 2009; Vazey and Aston-Jones, 2012; Kehagia *et al.*, 2014; Ye *et al.*, 2015) and serotonergic transmission (Harrison *et al.*, 1999; Fletcher *et al.*, 2007).

The primary source of forebrain noradrenaline is the locus coeruleus nucleus in the brainstem (Aston-Jones and Cohen, 2005), which is also an early site of pathology in Parkinson’s disease (Braak *et al.*, 2003). This suggests that the noradrenergic system is a potential target for therapeutic intervention. The drug atomoxetine is a noradrenaline reuptake inhibitor which increases noradrenaline levels in the prefrontal cortex (Bymaster *et al.*, 2002) and noradrenaline transporter occupancy (Seneca *et al.*, 2006). Atomoxetine facilitates attentional set-shifting in preclinical studies (Newman *et al.*, 2008) and improves response inhibition in preclinical models (Robinson *et al.*, 2008) and healthy humans via modulation of prefrontal cortex activity (Chamberlain *et al.*, 2009). In Parkinson’s disease, atomoxetine improves behavioural performance in a subgroup of patients, including enhanced response inhibition in relation to increased prefrontal cortex activity and fronto-striatal connectivity (Kehagia *et al.*, 2014; Ye *et al.*, 2015; Rae *et al.*, 2016).

Serotonin has also been implicated in the cognitive deficits associated with Parkinson’s disease, and executive functions mediated by the prefrontal cortex (Rubia *et al.*, 2005). The main source of forebrain serotonergic innervation is the raphe nuclei of the brainstem (Wilson and Molliver, 1991). This is also an early site of neurodegeneration in Parkinson’s disease (Braak *et al.*, 2003), which impairs serotonergic transmission in the prefrontal cortex (Politis *et al.*, 2010). Restoring serotonergic innervation is thus another potential target to improve the cognitive deficits in Parkinson’s disease. This notion is supported by a study showing that the serotonin reuptake inhibitor, citalopram, improves response inhibition in patients with moderate-to-severe disease, in association with increased prefrontal activation (Ye *et al.*, 2014).

Previous psychopharmacological imaging studies have assessed the effects of serotonin and noradrenaline reuptake inhibition on activity and connectivity during task performance. However, task-free (also known as resting-state) functional magnetic resonance imaging can be used to examine the effect of a drug on widespread brain network connectivity. Task-free functional magnetic resonance imaging allows for the inclusion of more diverse patients with significant cognitive and motor impairments while also minimizing task-related confounds, training demands and practice effects. It has been shown, for example, that noradrenaline influences fluctuations in brain network organization (van den Brink *et al.*, 2016, 2018), and atomoxetine enhances prefrontal cortical connectivity in Parkinson’s disease in proportion to its effect on verbal fluency, a marker of executive function (Borchert *et al.*, 2016).

The current study tested the hypotheses that the partial restoration of noradrenergic and serotonergic levels in Parkinson’s disease, via atomoxetine and citalopram respectively, restores brain network organization. To address this, we quantified the global and regional patterns of network integration and segregation using graph theoretical measures and compared these to indices of cognitive performance and disease severity. We tested two principal hypotheses: (i) Parkinson’s disease impairs whole-brain network function, quantified in terms of hub connectivity, modularity and centrality; and (ii) treatment by atomoxetine and citalopram restores these functional brain network properties, in a subset of patients according to severity and drug level.

## Materials and methods

### Participants

Thirty-three Parkinson’s disease patients were recruited from the Cambridge University Parkinson’s disease Research Clinic according to United Kingdom Parkinson’s disease Society Brain Bank criteria. Seventy-six age- and sex-matched controls were recruited from the healthy volunteers registered with the Cambridge University Parkinson’s disease Research Clinic and the Medical Research Council’s Cognition and Brain Sciences Unit. Inclusion criteria were (i) right-handed; (ii) age 45–80 years; (iii) non-demented clinically and with Mini-Mental State Examination score >26/30; (iv) no clinically significant current depression; (v) no history of significant psychiatric disorder or epilepsy; and (vi) no contraindications to magnetic resonance imaging, atomoxetine or citalopram. None of the patients declared symptoms of impulse control disorders. The study was approved by the local research ethics committee with exemption from clinical trials status by the United Kingdom Medicines for Human Use Regulatory Agency. All participants provided written consent.

Parkinson’s disease patients were administered the Unified Parkinson’s Disease Rating Scale motor subscale part III on each study day, and all participants underwent cognitive assessment using the Mini-Mental State Examination, digit span forward and backward, category and letter fluency (Rittman *et al.*, 2013), the revised Beck-Depression Inventory and a stop-signal reaction time task.

We anticipate that any potential future use of noradrenergic or serotonergic treatments targeting cognition in Parkinson’s disease would be adjunctive, and not an alternative, to standard dopaminergic therapy. Therefore, the effect of these drugs on patients was assessed in the context of their usual clinically optimized dopaminergic medication. Patients were not taking other directly serotonergic or noradrenergic medication, nor mono-amine-oxidase inhibitors. Levodopa equivalent dose was estimated (Tomlinson *et al.*, 2010) and included as a covariate in the analysis.

### Experimental design

A randomized double-blinded, placebo-controlled crossover design was used for patient treatment by atomoxetine, citalopram and placebo. Patients underwent three separate sessions, at least 6 days apart at approximately the same time of day, consisting of cognitive and neurological assessments and brain imaging. The drug order was counter-balanced using permutation within groups of six successive subjects to reduce session-order effects on the drug effect. One patient participated in the control and atomoxetine sessions but not the citalopram session. Each patient received a 40 mg oral dose of atomoxetine, a 30 mg oral dose of citalopram or a placebo capsule at the start of each session. Drug plasma concentrations were measured and patients were scanned 2 h after administration to coincide with peak plasma concentration for atomoxetine (Sauer *et al.*, 2005) and citalopram (Sangkuhl *et al.*, 2011). Controls were scanned once without drug or placebo to provide normative data. Note that the effect of disease (patient on placebo versus control) is confounded by potential placebo effects. The principal analysis of interest was the main effect of drug treatment within the patient group.

### Functional magnetic resonance imaging data acquisition and pre-processing

Task-free functional imaging was performed at rest using a TIM-Trio 3T magnetic resonance imaging scanner (Siemens Medical Systems, Erlangen, Germany). A minimum of 145 volumes were acquired using an echo-planar imaging sequence (repetition time 2000 ms, echo time 30 ms, matrix = 64 × 64, in-plane resolution of 3 × 3 mm, 32 slices of 3 mm thickness with a 0.75 mm interslice gap, and a flip angle of 78°). Structural Magnetization-Prepared Rapid Acquisition with Gradient Echo scans (repetition time of 2300 ms, echo time 2.86 ms, matrix = 192 × 192, in-plane resolution of 1.25 × 1.25 mm, 144 slices of 1.25 mm thickness, inversion time of 900 ms and flip angle of 9°) were also acquired during the same session.

In order to account for atrophy and in-scanner head movements, a pre-processing pipeline optimized for older subjects was used (Patel *et al.*, 2014). We used a study-specific template generated from the Magnetization-Prepared Rapid Acquisition with Gradient Echo images using the Diffeomorphic Anatomical Registration Through Exponentiated Lie Algebra algorithm (Ashburner, 2007). Structural images were segmented and grey and white matter images from all participants were iteratively warped together over six steps to create the study-specific template which was then affine transformed to Montreal Neurological Institute (MNI) space.

Functional images were pre-processed using a customized version of the brainwavelet toolbox. Pre-processing steps included removal of the first five volumes, coregistration of the mean echo-planar imaging image to the T1 image, transformation of the coregistered echo-planar imaging to MNI space using the flow fields generated by the Diffeomorphic Anatomical Registration Through Exponentiated Lie Algebra algorithm, slice-timing correction, combined regression of cerebrospinal fluid signal and motion derivatives, high-pass band filter (0.01 Hz) and wavelet despiking (Patel *et al.*, 2014).

We combined approaches to minimize in-scanner head movement-related effects on the blood oxygenation level dependent signal. Wavelet despiking was used to despike movement-related non-stationary events on a voxel level. Participants were excluded based on the average root mean-squared displacement computed from the translation parameters of head motion: average root mean-squared displacement over two standard deviations (SD) from the mean and/or 2 SDs from the mean difference between placebo and drug sessions for patients.

### Graph analysis

Graph theoretical analysis was used to investigate the characteristics of brain network organization. In the context of the brain, networks are composed of nodes, which represent brain regions, and edges, which represent the connectivity between the regions. Nodes were identified using a random 500-node parcellation to create nodes of approximately equal size. Nodes that were not sufficiently covered in ten participants or more were excluded (*n* = 27). Wavelet correlations were used to generate association matrices (Achard *et al.*, 2006). To generate binary graphs, local thresholds were applied (Alexander-Bloch *et al.*, 2010) with thresholds between 1% and 10%. An intermediate density of 6% was used for primary statistical analysis (Cope *et al.*, 2018). The graphs were Fisher-z transformed to normalize the correlation coefficients. A correction for multiple comparisons was not applied as network measures are not independent (Rittman *et al.*, 2019).

The Maybrain toolbox (https://github.com/RittmanResearch/maybrain) was used to compute the three main topological properties of the networks using the correlation matrices: (i) Path length is the average shortest path length in a network and provides a measure of the efficiency of network-wide communication. (ii) Clustering coefficient is a measure of the extent to which a node’s neighbours are inter-connected and provides insight into the local efficiency of a network. The organization of functional connectivity in the brain is similar to that of a small-world network which is characterized by low path length and high clustering coefficient, creating an efficient network architecture with low connection cost (Latora and Marchiori, 2003). (iii) Modularity reflects the functional divisions of brain networks into clusters which are densely intra-connected and more sparsely inter-connected.

### Hub metrics

Hubs are highly connected nodes and their function is vital for information processing in a small-world network (Heuvel and Sporns, 2013; Stam, 2014). The functional significance of these hubs was quantified by computing the number of nodes connected to the hub node (degree) and the extent to which the hub node is central to information processing within the network. Hub regions were defined as nodes with a connection strength over 1.5 SDs above the mean in a randomly selected cohort consisting of half of the control participants (*n* = 37). These nodes were used to compare hub metrics (degree, closeness centrality, betweenness centrality, Eigen centrality) in the other half of the controls, not used for hub identification (*n* = 38), to the patient placebo group (*n* = 30). Hub metrics were then compared in patients between no drug and drug sessions to determine if atomoxetine and/or citalopram modulated hub connectivity.

### Statistical analysis

Normative brain network graph metrics were quantified and compared between controls and the patient placebo group, using two-sample *t*-tests. Outlier participants beyond 2 SDs from the mean were removed prior to analysis. The effect of drug on network connectivity was investigated using a repeated-measures ANOVA for graph measures in patients. Measures of age, Unified Parkinson’s Disease Rating Scale motor subscale part III, levodopa equivalent dose (Tomlinson *et al.*, 2010), change in neuropsychological performance on drug versus no drug and drug plasma concentration were included as covariates to investigate interactions between patient demographic/clinical characteristics and the effects of atomoxetine and citalopram on network connectivity.

### Data availability

The terms of original participant consent prevent Open Access to raw data or other personally identifiable data but we would welcome requests from potential academic collaborators (please contact the senior author), while summary data and derived images may be requested from the corresponding author.

## Results

After exclusions, the data from 75 controls and 30 patients (atomoxetine condition: *n* = 30, citalopram condition *n* = 29) were carried forward for analysis. Patient demographic and neuropsychological information are shown in Table 1. Controls and patients were matched for sex, age and education. Relative to controls, patients had lower Mini-Mental State Examination and category fluency scores and longer stop-signal reaction times as expected.

**Table 1:** Participant clinical, cognitive and demographic characteristics at baseline before trial medication

	Patients mean (SD)	Controls mean (SD)	Difference (*P*-value)
Male:female	19:11	41:34	ns
Age (years)	67 (7.3)	67.1 (8.4)	ns
Education (years)	14.2 (3.6)	14.8 (4.0)	ns
Mini-Mental State Examination	28.4 (1.7)	29.2 (1.1)	0.009
Disease duration (years)	10.5 (4.4)		
Levodopa equivalent dose (mg/day)	870 (469)		
Unified Parkinson’s Disease Rating Scale motor subscale part III ‘on’	22.6 (6.8)		
Category fluency	18.3 (5.5)	24.3 (6.2)	0.0001
Letter fluency	16.0 (4.4)	18.3 (5.7)	ns
Digit span forward	7.0 (1.1)	7.3 (0.8)	ns
Digit span backward	5.5 (1.2)	6.0 (1.3)	ns
Stop-signal reaction time (ms)	198 (73)	164 (39)	0.02
Atomoxetine plasma concentration (ng/ml)	372.1 (167.4)		
Citalopram plasma concentration (ng/ml)	35.6 (14.7)		

Groups are compared by unpaired *t*-test or chi-squared test as appropriate.

Eleven nodes in the graph had a degree over 1.5 SDs from the mean in the randomly selected control cohort (*n* = 37) and were designated as hub nodes. The regions of these hub nodes are listed in Table 2, with numerical labelling according to the automated anatomical labelling atlas.

**Table 2:** Hub node regions used for analysis

Automated Anatomical Labeling atlas numerical label	Automated Anatomical Labeling atlas region
10	Right middle frontal gyrus, orbital part
16	Right inferior frontal gyrus, pars orbitalis
34	Right midcingulate area
55	Left fusiform gyrus
78	Right thalamus
81	Left superior temporal gyrus
83	Left superior temporal pole
84	Right superior temporal pole
90	Right inferior temporal gyrus
91	Left crus I of cerebellar hemisphere
100	Right lobule VI of cerebellar hemisphere

### Controls versus patients on placebo

There was no significant difference in path length or modularity between controls and patients on placebo. However, patients on placebo had lower clustering coefficient (*P* = 0.038), hub degree (*P* = 0.0001), hub betweenness centrality (*P* = 0.009), hub closeness centrality (*P* = 0.032) and hub Eigen centrality (*P* = 0.02).

### Effect of atomoxetine in patients

**Figure 1:**
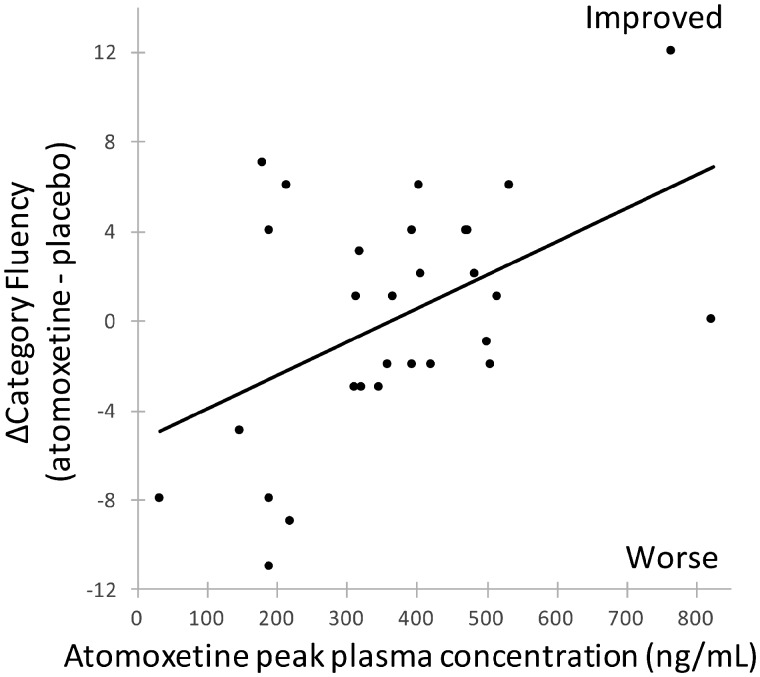
**Atomoxetine plasma concentrations.** Patients with higher peak plasma concentrations of atomoxetine demonstrated greater improvement in category fluency (measured by number of words produced in a category) on the drug relative to placebo (*P* = 0.006, *r*^2^ = 0.24).

Within the patient group, peak plasma concentration of atomoxetine correlated with change in category fluency on the drug; patients with higher plasma concentrations demonstrated greater improvement (*P* = 0.006, *r*^2^ = 0.24; Fig. 1).

**Figure 2:**
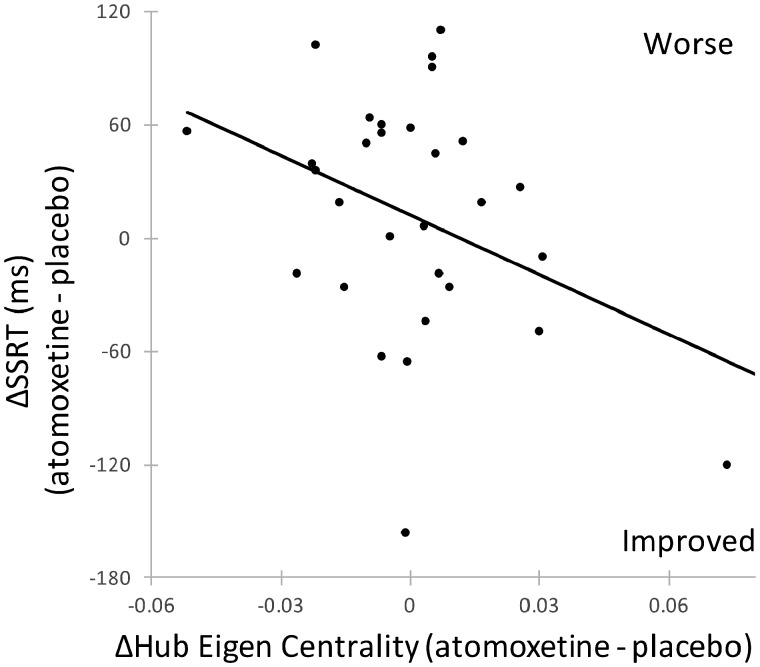
**Hub centrality on atomoxetine.** Patients with increased hub Eigen centrality on atomoxetine had faster stop-signal reaction times (ms) on the drug (*P* = 0.044, *r*^2^ = 0.14).

There was no significant main effect of atomoxetine on global clustering coefficient, path length, or modularity, relative to placebo. There was also no interaction between the effect of atomoxetine on these global graph metrics and covariates of interest. Lower baseline hub metrics correlated with a greater increase in the respective metric with atomoxetine towards controls for hub betweenness centrality (*P* = 0.022, *r*^2^ = 0.17), hub closeness centrality (*P* < 0.0005, *r*^2^ = 0.47) and hub Eigen centrality (*P* = 0.01, *r*^2^ = 0.21). Behavioural inhibition on a stop-signal task improved in patients on atomoxetine in proportion to baseline Eigen centrality (*P* = 0.025, *r*^2^ = 0.17) and increased hub Eigen centrality on the drug (*P* = 0.044, *r*^2^ = 0.14) (Fig. 2).

### Effect of citalopram in patients

**Figure 3:**
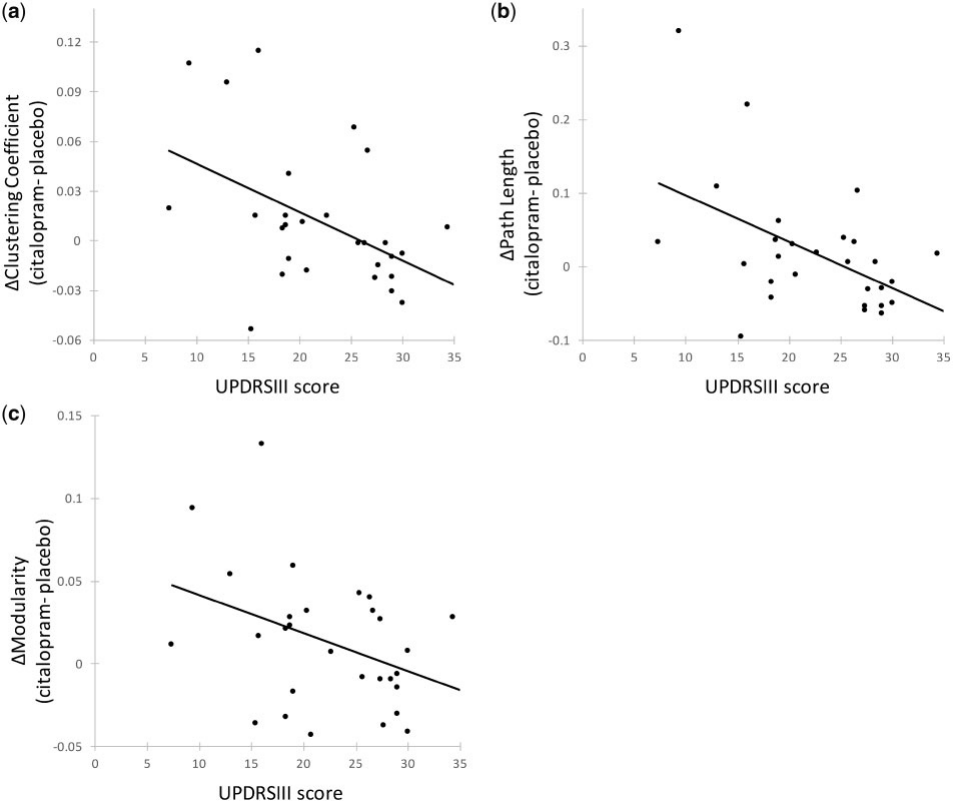
**Network connectivity on citalopram.** Citalopram decreased (**A**) clustering coefficient (*P* = 0.01, *r*^2^  =  0.22), (**B**) path length (*P* = 0.006, *r*^2^ = 0.25) and (**C**) modularity (*P* = 0.043, *r*^2^ = 0.14) in Parkinson’s disease patients with higher Unified Parkinson’s Disease Rating Scale motor subscale part III scores.

Citalopram did not change fluency or inhibitory control at the group level or in proportion to plasma concentration. Lower baseline graph metrics correlated with a greater increase on citalopram towards controls in the respective metric for clustering coefficient (*P* = 0.031, *r*^2^ = 0.16), modularity (*P* = 0.036, *r*^2^ = 0.15), hub degree (*P* < 0.0005, *r*^2^ = 0.59), hub betweenness centrality (*P* < 0.0005, *r*^2^ = 0.76) and hub closeness centrality (*P* < 0.0005, *r*^2^ = 0.57). Citalopram decreased clustering coefficient (*P* = 0.01, *r*^2^ = 0.22), path length (*P* = 0.006, *r*^2^ = 0.25) and modularity (*P* = 0.043, *r*^2^ = 0.14; Fig. 3) in proportion to disease severity (higher Unified Parkinson’s Disease Rating Scale motor subscale part III scores). Citalopram did not alter group-wise global or hub metrics.

## Discussion

This study reinforces the potential for noradrenergic reuptake inhibition (by atomoxetine) to improve brain network function in Parkinson’s disease, aimed at improving executive function. The potential for a serotonergic therapeutic effect depends on the severity of disease, as measured clinically by the Unified Parkinson’s Disease Rating Scale motor subscale part III. These drug effects are set in the context of the impact of Parkinson’s disease on brain network function including the reduction of hub connectivity, and network modularity and centrality.

The task-free functional magnetic resonance imaging encompassed both motor and cognitive networks. Although the loss of task-specificity may be seen as a disadvantage, the task-free approach has many advantages, including the minimization of performance confounds and practice effects in crossover designs; the scalability across severity levels, sites and languages in multicentre studies; and quantification of connectivity in nodes across several networks. It has revealed commonalities across many degenerative disorders in terms of network reorganization and hub connectivity in particular (Crossley *et al.*, 2014). This makes it well suited to study a heterogeneous disorder such as Parkinson’s disease, with its motor and cognitive impairments (Williams-Gray *et al.*, 2013; Yarnall *et al.*, 2014).

In addition to the intrinsic heterogeneity of Parkinson’s disease, an oral dose of drug leads to widely differing plasma levels between individuals (Ye *et al.*, 2015). This means that a single dose may not be equally effective in all patients and simple group-wise comparisons are likely to produce null findings. This is also true for dopaminergic effects on non-motor functions (Rowe *et al.*, 2008; Cools *et al.*, 2009) but it places particular emphasis on the need for predictive models of response, or at least stratification tools for future clinical trials. In this exploratory study however, we sought to explain changes in the effect of drug in relation to drug levels and disease severity.

For atomoxetine, the change in fluency correlated with drug plasma concentration. Previous authors have suggested the relationship between atomoxetine concentration and task performance follows a Yerkes-Dodson ‘inverted U-shaped’ function (Aston-Jones and Cohen, 2005; Bari and Aston-Jones, 2013) which is influenced by baseline noradrenergic levels. For example, an individual with relatively higher baseline noradrenergic levels may overshoot the range for optimal performance if treated with atomoxetine (Housden *et al.*, 2011). Atomoxetine administration in mild disease with presumed relatively intact endogenous noradrenaline systems was detrimental, indicative of an ‘overdose’, placing noradrenaline beyond its optimal range (Ye *et al.*, 2015). Here, we examined the effect of atomoxetine on verbal fluency, as both a marker of executive function and as a significant predictor of cognitive decline (Williams-Gray *et al.*, 2007). Atomoxetine plasma concentration and the change in fluency were correlated. A similar linear relationship was identified between atomoxetine plasma concentration and improvement on a task of problem-solving and working memory (Kehagia *et al.*, 2014). We did not observe a direct relationship between atomoxetine plasma concentration and inhibitory control (the stop-signal reaction time task). However, there was an indirect relationship, expressed in terms of the effect of atomoxetine on hub Eigen centrality and the effect of atomoxetine on inhibitory control. The effects of atomoxetine on different cognitive domains may be a feature of their different noradrenergic ‘optima’ or the differential degrees of neurodegeneration in neural circuits serving each cognitive domain (Haber, 2003; Rowe *et al.*, 2008; Rowe and Rittman, 2016). In addition to plasma concentration, disease severity and the extent of noradrenergic denervation also influence response to atomoxetine in these patients. Noradrenaline is significantly reduced in the brain of Parkinson’s disease patients (Goldstein *et al.*, 2014) and correlates with motor disease severity (Marié *et al.*, 1995). Previous work has demonstrated that the effect of atomoxetine on fronto-striatal effective connectivity (Rae *et al.*, 2016) and stop-related inferior frontal gyrus activation (Ye *et al.*, 2015) depends on disease severity. Hence, we suggest that both plasma concentration and noradrenergic denervation, indexed by disease severity, contribute to drug response in these patients. 

Considering the widespread noradrenergic and serotonergic projections which are compromised in Parkinson’s disease, we predicted an effect of atomoxetine and citalopram on global network topology. Highly connected hub regions are preferentially affected in Parkinson’s disease, as well as other neurodegenerative diseases (Crossley *et al.*, 2014), and loss of hub connectivity, particularly in the prefrontal cortex, has been linked to executive dysfunction (Rittman *et al.*, 2016). Although atomoxetine did not significantly alter whole-brain graph measures, it did change hub connectivity. Patients with increased hub Eigen centrality on atomoxetine demonstrated improved inhibitory control in terms of the stop-signal task performance recorded outside the scanner. These improvements in behavioural inhibition and hub Eigen centrality on atomoxetine were seen in patients with lower baseline hub Eigen centrality on placebo. The stop-signal task elicits activation of the prefrontal cortex, particularly the right inferior frontal gyrus and pre-supplementary motor area (Garavan *et al.*, 1999; Aron *et al.*, 2003; Rubia *et al.*, 2003; Eagle *et al.*, 2008; Rae *et al.*, 2015). Improved inhibitory control following atomoxetine administration is mediated by inferior frontal gyrus activity and its connectivity through the basal ganglia in Parkinson’s disease (Ye *et al.*, 2015; Borchert *et al.*, 2016; Rae *et al.*, 2016) and healthy adults (Chamberlain *et al.*, 2009). In our study, the change in connectivity of hubs correlated with improved stop-signal reaction time task on atomoxetine: these hub regions overlapped with those known to associate with response inhibition.

Previous studies of citalopram in healthy volunteers found reduced task-free functional connectivity following drug administration (McCabe and Mishor, 2011; Schaefer *et al.*, 2014; Klaassens *et al.*, 2017). In the context of depression, these authors hypothesized that citalopram acted by normalizing functional connectivity. In Parkinson’s disease there is also a disruption of serotonergic transmission (Politis and Niccolini, 2015) which progresses over time (Politis *et al.*, 2010), including regions of prefrontal cortex that are closely associated with inhibitory control, learning and cognitive flexibility, not only affective cognition. For example, the behavioural effects of serotonergic manipulations on response inhibition are mediated by the prefrontal cortex (Del-Ben *et al.*, 2005; Macoveanu *et al.*, 2013). Here, we found that improved clustering coefficient, modularity and hub connectivity on citalopram were associated with lower baseline values for these metrics. However, these measures were not associated with any change in cognitive performance on the drug. We previously reported that the benefit of citalopram on response inhibition and an associated neural activation emerged only in patients with more advanced disease, especially if they had relatively preserved fronto-striatal connectivity (Ye *et al.*, 2014). In the current analysis, the effect of Parkinson’s disease was to reduce the brain’s clustering coefficient and centrality measures. If this were primarily due to serotonergic impairments, we would expect that citalopram would increase these network properties towards a normal level with more severe disease. However, despite a modest positive effect on connectivity in patients with mild disease, this effect declined with disease severity. In other words, patients with more severe disease appeared resistant to the effect of citalopram on the topology of brain network organization. This was unexpected and could be the result of altered response to citalopram due to significant degeneration in the more severely affected patients. Serotonergic transmission is disrupted in Parkinson’s disease and the extent of denervation progresses with disease severity (Politis *et al.*, 2010; Politis and Niccolini, 2015). An impaired serotonergic architecture could explain the resilience of global and hub metrics to citalopram in more advanced disease. We speculate that our findings result from the heterogeneity of the effects of Parkinson’s disease and variable sites of action of citalopram, leading to a null effect on global measures in patients with severe disease, in contrast to the previously reported effects on focal prefrontal cortical activations (Ye *et al.*, 2014). Future investigations using 7T-magnetic resonance imaging of the locus coeruleus (Betts *et al.*, 2019), PET imaging of serotonergic receptor density or noradrenaline transporter density, or dopamine transporter imaging using single-photon emission computed tomography, could provide further insight into the relationship between drug response and the integrity of noradrenergic, dopaminergic and serotonergic systems.

This study had several limitations. First, although patients received one dose of atomoxetine or citalopram during brain imaging, chronic drug administration may differentially affect cognitive and functional connectivity (Koda *et al.*, 2010). This could be mediated by down-regulation of neurotransmitter receptors or synthesis and future studies should investigate the effect of chronic drug treatment. Second, blood oxygenation level dependent signals could have been influenced by atomoxetine, citalopram and/or dopaminergic drugs. However, this is unlikely as cerebral blood flow remains normal following atomoxetine (Marquand *et al.*, 2012) and citalopram (Macoveanu *et al.*, 2013) administration, and dopaminergic therapy was kept constant in the within-subject crossover analyses. Although the non-trial medication was kept constant as part of patients’ standard therapy in the drug versus placebo comparison, this was not controlled for when comparing patients on placebo to healthy controls. Indirect effects of placebo treatment on cognitive or neurotransmitter systems confound the comparison of patients with controls in this study. However, this caveat does not affect the comparison of drug versus placebo in patients. Third, the mediating effect of atomoxetine on functional connectivity has several potential contributory mechanisms. Atomoxetine can affect dopaminergic as well as noradrenergic transmission (Bymaster *et al.*, 2002). However, effects of atomoxetine on response inhibition have been shown to be primarily mediated by noradrenergic transmission in animal studies (Bari *et al.*, 2009).

In conclusion, atomoxetine and citalopram modulate resting-state functional connectivity in Parkinson’s disease in different ways. We suggest that hub connectivity mediates the effect of atomoxetine on executive function while citalopram alters whole-brain graph metrics according to disease severity. This study provides support for the use of task-free imaging methods to assess the impact of drugs on neurocognitive systems in patients.

## Abbreviations

SD =: Standard deviation
